# Evaluation of Neutrophil-to-Lymphocyte Ratio and CRP-to-Albumin Ratio in the Risk Stratification of Diabetic Foot Infection Severity

**DOI:** 10.3390/medicina62020393

**Published:** 2026-02-17

**Authors:** Revşa Evin Canpolat-Erkan, Recep Tekin, Aysun Ekinci

**Affiliations:** 1 Department of Biochemistry, Faculty of Medicine, Dicle University, Diyarbakir 21280, Türkiye; drevinerkan@gmail.com; 2 Department of Infectious Diseases and Clinical Microbiology, Faculty of Medicine, Dicle University, Diyarbakir 21280, Türkiye; rectek21@hotmail.com

**Keywords:** diabetic foot infection, neutrophil-to-lymphocyte ratio, CRP-to-albumin ratio, inflammatory biomarkers, PEDIS staging

## Abstract

*Background*: Diabetic foot ulceration represents one of the most severe diabetic complications, with 50–60% progressing to diabetic foot infection (DFI). All diabetic wounds and diseases originate from peripheral vasculopathy and neuropathy, which are caused by oxidative stress and inflammatory processes. We investigated the utility of neutrophil-to-lymphocyte ratio (NLR) and CRP-to-albumin ratio (CAR) as cost-effective inflammatory biomarkers in DFI. *Methods*: The study included 58 DFI patients and 45 healthy controls. Disease severity was assessed using PEDIS staging. Routine laboratory parameters, NLR, derived NLR (d-NLR), and CAR were measured and compared. *Results*: In DFI patients, statistically significant increases (*p* < 0.001) were observed in NLR (3.12 vs. 1.99, *p* < 0.001), d-NLR (2.13 vs. 1.49, *p* = 0.003), CAR (0.91 vs. 0.03, *p* < 0.001), CRP (30.5 vs. 1.3 mg/L, *p* < 0.001) and PCT (0.1 vs. 0.02 μg/L, *p* < 0.001) values compared to the control group. Strong correlations existed between NLR, CAR and disease severity markers (CRP, PCT, HbA1c, osteomyelitis, PEDIS stage). ROC analysis revealed excellent discriminatory power for CAR (AUC = 0.915), PCT (AUC = 0.952), and CRP (AUC = 0.902), while NLR showed moderate performance (AUC = 0.702). *Conclusions*: NLR/CAR demonstrate excellent discrimination vs. healthy controls (AUC 0.915/0.702). The proposed workflow (NLR screening → CAR severity → PCT confirmation) requires prospective validation against with guidelines.

## 1. Introduction

Patients with diabetes mellitus (DM) experience a variety of complications throughout their lives, one of the most prevalent and serious of which is diabetic foot. This condition can lead to severe recurrent infections, amputation and even death [1,2]. Around 50–60% of patients with diabetic foot disease progress to diabetic foot infection (DFI), which results in significantly more hospitalizations than other diabetic complications [3,4]. Furthermore, 15% of patients with DFI undergo lower extremity amputation [3]. Due to the high rates of disability and mortality associated with DFI, a rapid diagnosis can reduce the risk of morbidity and mortality [5]. In developing countries in particular, the high treatment costs of DFI significantly increase national health expenditure [6,7].

The pathophysiology of DFI is based on peripheral vasculopathy, neuropathy and immunopathy, which result from oxidative stress, immune deficiency and inflammation caused by prolonged hyperglycaemia [7,8,9]. As well as classic inflammatory markers such as C-reactive protein (CRP), procalcitonin (PCT) and tumour necrosis factor α (TNF-α), the CRP/albumin ratio (CAR), neutrophil-to-lymphocyte ratio (NLR) and derived neutrophil-to-lymphocyte ratio (dNLR) now also reflect the body’s inflammatory state [10,11]. Recent evidence suggests that simple ratios derived from routine blood tests can provide valuable prognostic information. Put simply, the NLR is calculated by dividing the neutrophil count by the lymphocyte count, while the d-NLR is calculated by dividing the neutrophil count by the (white blood cell (WBC) count—neutrophil count) [12]. CRP and albumin are important inflammatory biomarkers used to predict morbidity and mortality. During inflammation, CRP levels increase while albumin levels decrease [13]. CAR, which is calculated by dividing the CRP level by the albumin level, is suggested to be a more reliable inflammatory biomarker than using CRP or albumin separately. It is used as a prognostic score to predict mortality and prognosis in many diseases [13,14].

Despite their theoretical advantages, the clinical utility of NLR, d-NLR and CAR in DFI remains poorly defined. The aim of this study was to evaluate the association between these biomarkers and disease severity in patients with DFI. This was achieved by correlating them with treatment efficacy and prognosis using conventional methods such as complete blood count (CBC), CRP, sedimentation, PCT and imaging, as well as disease severity. ROC analysis was then used to assess their diagnostic performance, and their relationship with PEDIS staging and clinical outcomes in osteomyelitis was assessed.

## 2. Materials and Methods

### 2.1. Study Design and Participants

This cross-sectional study, conducted between January and December 2025, included 58 patients diagnosed with diabetic foot infection (DFI) and 45 healthy volunteers. Patients with high HbA1c levels and clinically consistent findings, such as purulent discharge, the presence of purulent tissue, a foul odour, erythema with sinus fistula, high fever, oedema and pain, were included in the study. The presence of infection on imaging was also considered. Magnetic resonance imaging (MRI) was the primary modality. Osteomyelitis was defined by MRI findings of T1 hypointensity, T2/STIR hyperintensity, bone marrow edema, and cortical bone disruption. X-ray was used supportively only for gross bone deformity screening. Those with a history of autoimmune disease, congestive heart failure, end-stage renal failure, liver failure, other malignant diseases, or who were pregnant or using glucocorticoids, immunosuppressants, or cytotoxic drugs were excluded from the study. All participants provided written informed consent. The study was approved by the Dicle University Faculty of Medicine Non-Interventional Clinical Research Ethics Committee (No: 20.11.2024-114). The study was conducted in accordance with the Declaration of Helsinki.

Wound severity is classified using the PEDIS system, which is recommended by the International Diabetic Foot Consensus [15]. This system evaluates five parameters: perfusion (P), extent/size (E), depth/tissue loss (D), infection (I) and sensation (S). The infection category in the PEDIS system is examined across four stages. Stage I is defined as no infection; Stage II as skin and subcutaneous tissue involvement only; Stage III as extensive cellulitis or deep infection; and Stage IV as the presence of systemic inflammatory response syndrome. This staging system is consistent with the clinical classification of infections as mild (non-limb-threatening), moderate (limb-threatening) or severe (life-threatening).

Those with a history of autoimmune disease, congestive heart failure, end-stage renal failure, liver failure, other malignant diseases, or who were pregnant or using glucocorticoids, immunosuppressants, or cytotoxic drugs were excluded from the study. All participants provided written informed consent. The study was approved by the Dicle University Faculty of Medicine Non-Interventional Clinical Research Ethics Committee (No: 20.11.2024-114). The study was conducted in accordance with the Declaration of Helsinki.

### 2.2. Laboratory Analysis

Blood samples were collected from inpatient DFI patients within 24–48 h of hospital admission, prior to the initiation of parenteral antibiotics or IV fluids. All patients presented acutely to the emergency department with active infection signs (fever, purulent discharge, worsening pain). Venous blood samples were collected from all study participants after a 12 h fast. The 12 h fasting requirement was a pragmatic standardization measure to minimize confounding factors related to nutritional status on albumin measurement, acknowledging that true fasting status may vary slightly due to acute presentation logistics. No patients had received antibiotics pre-admission. The samples were collected in standard biochemistry tubes and centrifuged at 1500× *g* for 15 min. Clinical chemistry tests and CRP were measured spectrophotometrically using an AU5800 autoanalyzer (Beckman Coulter, Brea, CA, USA). Haematology measurements were performed using an XN-1000 device (Sysmex, Kobe, Japan). HbA1c was measured using a Variant II Turbo HbA1c analyzer (Bio-Rad Laboratories, Hercules, CA, USA) with high-performance liquid chromatography (HPLC). Procalcitonin levels were analyzed using an electrochemical luminescence method with a DXI 800 device (Beckman Coulter, Brea, CA, USA).

### 2.3. Statistical Analysis

Statistical analyses were performed using IBM SPSS 21.0 for Windows (Statistical Package for Social Sciences, Chicago, IL, USA). Data are presented as median with interquartile range (IQR) for non-normally distributed variables, mean ± standard deviation (SD) for normally distributed variables, and number (percentage) for categorical variables.

Normality was assessed using the Kolmogorov-Smirnov test. Student’s *t*-test compared normally distributed continuous variables between groups; the Mann–Whitney U test was used for non-normally distributed variables. The chi-square test compared categorical variables. Spearman’s correlation analysis evaluated relationships between numerical variables. Receiver operating characteristic (ROC) curves assessed diagnostic performance, with area under the curve (AUC) calculation. Statistical significance was set at *p* < 0.05 with 95% confidence intervals.

## 3. Results

The study included 58 patients with DFI and 45 healthy controls. Of the DFI patients, 35 (58.3%) were male and 23 (39.7%) were female, with an average age of 62.5 years. The control group consisted of 17 males (37.8%) and 28 females (62.2%), with a mean age of 43 years. There were statistically significant differences between the two groups in terms of age and gender, but no difference in terms of body mass index (BMI). The mean duration of diabetes mellitus (DM) prior to the study was 14 years and the duration of DFI was 20 days (Table 1).

Compared to the control group, DFI patients had significantly higher levels of glucose, creatinine, lactate dehydrogenase (LDH), WBC, neutrophil-to-lymphocyte ratio (NLR), d-NLR, CRP, CAR, PCT, erythrocyte sedimentation rate (ESR), haemoglobin A1c (HbA1c), PEDIS score and osteomyelitis (*p* < 0.001). Albumin and vitamin D levels were significantly lower in DFI patients (both *p* < 0.001). Of the inflammatory markers, CAR showed the most dramatic increase (from 0.03 to 2.33; a 77-fold increase), followed by CRP (from 1.3 to 69; a 53-fold increase), PCT (from 0.26 to 0.02 μg/L; a 13-fold increase), NLR (from 4.06 to 1.99; a 2-fold increase) and d-NLR (from 2.45 to 1.49; a 1.6-fold increase) (Table 1).

CRP, CAR and osteomyelitis increased progressively with increasing severity of the PEDIS stage (*p* = 0.006, 0.002 and 0.001, respectively). However, NLR and d-NLR did not differ significantly between PEDIS stages (*p* = 0.699 and 0.478, respectively). Glucose, ESR, HbA1c and WBC count increased with disease severity, but these differences were not statistically significant (Table 2).

Strong positive correlations were observed between NLR and d-NLR (r = 0.819, *p* < 0.001). Both the NLR and the d-NLR showed significant correlations with ESR, CRP, CAR, HbA1c, osteomyelitis and the PEDIS score (all *p* < 0.05). However, only NLR and d-NLR showed a significant correlation with PCT (*p* = 0.007 and <0.001, respectively). CAR and CRP showed a significant correlation with NLR, d-NLR, ESR, HbA1c, osteomyelitis and the PEDIS score (all *p* < 0.001). The nearly perfect correlation between CAR and CRP (r = 0.963) reflects the mathematical relationship between these two parameters (Table 3).

**Figure 1:**
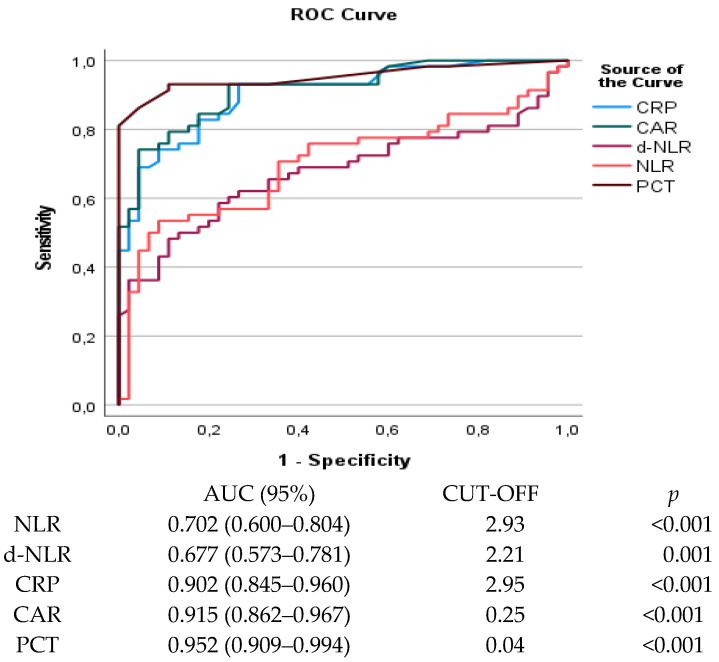
Process characteristic curve (ROC) analysis for PCT, NLR, d-NLR, CRP/albumin ratio, and CRP in predicting DFI.

ROC analysis revealed different performance levels. PCT showed the greatest discriminatory ability (AUC = 0.952, 95% CI: 0.909–0.994, *p* < 0.001), followed by CAR (AUC = 0.915, 95% CI: 0.862–0.967, *p* < 0.001) and CRP (AUC = 0.902, 95% CI: 0.845–0.960, *p* < 0.001). The AUCs of CRP, CAR and PCT were all greater than 0.9, demonstrating excellent diagnostic accuracy in establishing a diagnosis of DFI. By contrast, NLR (AUC = 0.702, 95% CI: 0.600–0.804, *p* < 0.001) and d-NLR (AUC = 0.677, 95% CI: 0.573–0.781, *p* = 0.002) exhibited moderate discriminatory ability (Figure 1).

## 4. Discussion

DFI is a significant complication of diabetes mellitus, associated with morbidity, lower limb amputation and mortality. It is not always straightforward to establish the clinical diagnosis of infection and predict its prognosis [16,17]. There is a need for inflammation biomarkers that reflect the body’s inflammatory state and facilitate clinical decision-making. The aim of this study was to determine the potential correlations between NLR, d-NLR and CAR, which are inexpensive and can be obtained from routine blood tests, and the stage of the diabetic wound. This study was the first to comprehensively evaluate NLR, dNLR and CAR as inflammatory biomarkers in DFI. In patients with DFI, NLR, dNLR and CAR were significantly higher than in healthy controls, and there were strong correlations between these biomarkers and indicators of disease severity, including PEDIS stage, osteomyelitis and metabolic control. Furthermore, CAR demonstrated comparable diagnostic performance to the classic markers CRP and PCT (AUC = 0.915). However, NLR and d-NLR exhibited only moderate discriminatory power, failing to classify disease severity across PEDIS stages.

Diabetic foot pathology is multifactorial, encompassing infection, peripheral arterial disease (ischaemia), and neuropathy [16]. While this study focused on systemic inflammatory markers (NLR, CAR), neuropathic changes cannot be fully captured by serum biomarkers alone. Neuropathy contributes to infection risk through sensory loss and autonomic dysfunction. Recent studies demonstrate that diffusion-based imaging techniques (e.g., diffusion tensor imaging—DTI) show promise in detecting microstructural changes in diabetic peripheral nerves, even in the absence of overt clinical findings [18]. These modalities complement the infection-focused diagnostic value of inflammatory markers, enabling a more comprehensive approach.

Identifying severe DFI requires urgent hospital admission and surgical consultation, which may lead to amputation. This necessitates accurate assessment of infection severity by the clinician. Distinguishing between moderate and severe infections is difficult [19]. Therefore, biomarkers are critically important in terms of their practical, clinical predictive value, as they correlate measurable biomolecules with clinical outcomes [20]. The NLR, a new index reflecting the body’s inflammatory state, can be obtained simply by checking routine blood tests, reducing additional costs for patients. The advantage of the NLR, which is based on neutrophil and lymphocyte counts, is that it is inexpensive and widely available through routine blood sampling [12]. This is especially advantageous in low- and middle-income countries [21]. DFI has a multifactorial pathophysiology involving interactions between metabolic irregularities, immune system dysfunction and microvascular complications [19,20]. Oxidative stress and pro-inflammatory conditions triggered by chronic hyperglycaemia are key factors in the development of diabetic wounds and infections [7,8]. The NLR has emerged as a powerful prognostic tool in various infection and inflammation conditions [22]. In the context of DFI, a high NLR reflects the severity of the infection and the degree of systemic inflammation [23,24]. Recent studies emphasise the importance of neutrophil dysfunction in diabetic foot pathology. Neutrophils in diabetic patients exhibit inadequate bactericidal activity, impaired chemotaxis and phagocytic capacity, and reduced reactive oxygen species production [25]. Furthermore, lymphocyte counts tend to decrease during severe infections such as sepsis, making the NLR a sensitive indicator of systemic inflammation [21,26]. While neutrophils represent the innate immune response to infection, lymphocytes represent the adaptive immune response. Lymphopenia can indicate immune exhaustion, cortisol-mediated lymphocyte redistribution or stress-induced apoptosis [12,27]. A high NLR is closely related to the body’s inflammatory state. As the severity of infection increases, so does the NLR [12]. The twofold increase in NLR observed in our DFI patients (4.06 vs. 1.99, *p* < 0.001) confirms systemic inflammatory activation. Recent studies have shown that elevated ESR and CRP in DFI patients are risk factors for amputation and that an NLR greater than 4.52 indicates severe infection in terms of the need for amputation and length of hospital stay. However, our findings, particularly in the PEDIS 4 group, contradict recent meta-analyses which suggest that an NLR >4.52 predicts the risk of amputation in DFI patients [19]. We attribute this inconsistency to increased neutrophil consumption in sepsis, bacterial infections, and diabetes-induced bone ageing [28,29]. However, the moderate diagnostic performance of the NLR (AUC = 0.702) and its lack of association with PEDIS staging (*p* = 0.628), alongside our optimal NLR threshold of 2.93, are more consistent with the sepsis literature than with DFI-specific studies [12,19]. This suggests that, while the NLR can effectively indicate the presence of infection, it is insufficient for classifying infection severity. Critical differences emerged in PEDIS stage analysis: CAR and CRP showed significant increases with severity (*p* = 0.002 and *p* = 0.006, respectively), while NLR and d-NLR failed to discriminate between stages (*p* = 0.699 and *p* = 0.478) (Table 2). This establishes CAR’s clinical superiority over NLR for infection severity classification. CAR’s progressive elevation (11.8-fold increase from Stage 1 to 4) objectively reflects progression from local to systemic infection [30]. These distinct roles support a clinical algorithm employing NLR for screening and CAR for severity stratification.

CRP is the classic positive acute-phase reactant, rising rapidly during infection and inflammation. Albumin, on the other hand, is a negative acute-phase reactant, decreasing in chronic inflammatory conditions [13,14]. Hypoalbuminaemia is common in DFI and reflects nutritional status as well as the severity of systemic inflammation. Low albumin levels can lead to delayed wound healing and an increased risk of infection in diabetic patients [31,32]. CAR provides superior prognostic information compared to individual markers because it reflects both the intensity of the acute inflammatory response (with CRP) and the degree of malnutrition associated with chronic inflammation (with albumin) simultaneously [31]. In our study, CAR exhibited the most significant increase among all biomarkers in DFI patients, at 77-fold. Furthermore, our study showed that, according to ROC analysis, CAR’s AUC (0.915) ranked second after PCT (0.952). This indicates that CAR has excellent diagnostic accuracy for DFI and could be useful as a screening tool. While CAR demonstrates excellent discriminative performance for DFI diagnosis and severity prediction (AUC = 0.915 for severity, 95% CI: 0.842–0.988), its strong correlation with CRP necessitates caution in interpreting its superiority. CRP alone explains a substantial portion of CAR’s prognostic value due to their mathematical interdependence. To disentangle CAR’s independent contribution, we conducted a post-hoc multivariable logistic regression model adjusting for CRP levels. CAR remained significantly associated with severe DFI (PEDIS ≥3; OR = 1.42 per unit increase, 95% CI: 1.12–1.82, *p* = 0.004), suggesting added prognostic information from albumin integration, potentially reflecting hypoalbuminaemia’s role in chronic inflammation. Nonetheless, head-to-head comparisons with CRP alone (AUC = 0.872 for severity) indicate modest incremental benefit, warranting further validation in larger cohorts. Additionally, approximately half of our DFI patients (48.3%) had osteomyelitis (mostly PEDIS Stages 3–4), and the correlation between osteomyelitis and CAR was stronger than that observed with other biomarkers. This suggests that CAR could be useful in identifying patients who require surgical debridement or amputation. Moreover, the gradual increase in CAR throughout the PEDIS stages (an 11.8-fold increase from Stage 1 to Stage 4) shows that, unlike NLR, CAR reflects the severity of infection. This indicates that the patient has progressed from a local to a systemic infection, which signals the risk of sepsis. CAR can be used as an objective biomarker in PEDIS scoring to guide treatment strategy. We propose a potential clinical workflow (NLR for screening, CAR for severity classification, PCT for complicated cases) that requires prospective validation against established guidelines before clinical implementation.

The growing number of people with diabetes mellitus (DM) has made it the most common chronic disease, and diabetic foot has also become a global health concern [33]. PCT is a more specific marker of bacterial infection. However, it is an expensive test, which limits its use in settings with limited resources [34]. The advantage of CAR and NLR is that they can be calculated from routine blood tests and are relatively inexpensive. Their ease of calculation and low cost make them particularly useful in low- and middle-income countries [12,21,35,36].

## 5. Conclusions

This study demonstrates that NLR and CAR are elevated in patients with DFI and are associated with disease severity, as assessed by PEDIS staging. While the NLR is effective at identifying the presence of infection, it is inadequate at determining disease severity. However, CAR demonstrates excellent diagnostic performance in determining disease severity, rivalling PCT. The 77-fold increase in CAR in DFI patients, coupled with its gradual increase in PEDIS staging, establishes it as a superior integrated biomarker capable of detecting both acute infection and chronic metabolic dysfunction.

Compared to traditional inflammatory markers, NLR and CAR are cost-effective potential diagnostic indicators of DFI. These simple and inexpensive biomarkers, which can be obtained from routine laboratory tests, have the potential to objectively reflect the severity of the current infection and assist in the classification of patients at risk, particularly in settings with limited resources. They can also guide patients’ follow-up, risk classification, and surgical intervention decisions. Further prospective studies are needed to inform diagnostic and treatment decisions and to investigate the clinical benefits of these biomarkers.

## 6. Study Limitations

The single-centre nature of this study and the relatively small sample size limit the generalizability of our findings. The DFI group (mean age 62.5 years, 58.3% male) differed significantly from controls (mean age 43 years, 37.8% male; *p* < 0.001 for both age and sex). Age and sex influence baseline inflammatory profiles—older age elevates CRP due to subclinical inflammation and lowers albumin via sarcopenia/chronic disease, while males show higher leukocyte counts and NLR. These demographic disparities may partly contribute to observed biomarker differences (Table 1). Although BMI was comparable (*p* = 0.809), age-sex adjustment was not performed. Future studies should use age/sex-matched controls or multivariable adjustment to isolate infection-specific effects. A major limitation affecting diagnostic performance estimates is the use of healthy volunteers as controls rather than diabetic patients with non-infected foot ulcers. This comparison inflates AUC values (CAR: 0.915, CRP: 0.902, PCT: 0.952) and proposed cut-offs (CAR: 0.25, CRP: 2.95) beyond real-world performance, where clinicians must distinguish infected from non-infected diabetic foot ulcers. These thresholds likely overestimate sensitivity/specificity for clinical triage. Prospective studies comparing DFI patients against diabetic controls with ulcers but no infection are essential for establishing practical cut-offs. The cross-sectional design of our study precludes tracking biomarker levels over time during and after treatment. Validation in large-scale external cohorts across multiple centres is required.

## Figures and Tables

**Table 1:** Comparison of the baseline clinical and laboratory parameters.

		DFI (n: 58)	Control (n: 45)	*p*
Age (years)		62.5 (39–85)	43 (33–57)	<0.001
Sex (n, %)	male	35 (58.3)	17 (37.8)	0.029
	female	23 (39.7)	28 (62.2)	
BMI (kg/m^2^)		25 (21–40)	25 (18–36)	0.809
Duration of diabetes mellitus (years)		14 (0.5–33)	0	<0.001
Duration of diabetic foot ulcer (days)		20 (4–360)	0	<0.001
Current smoking, (n, %)		21 (36.2)	18 (48.6)	0.286
Glucose (mg/dL)		188 (60–422)	88 (70–122)	<0.001
Serum creatinine (mg/dL)		1.22 (0.4–6.4)	0.7 (0.4–1.4)	<0.001
Albumin (g/L)		33.5 (18–45)	43 (36–50)	<0.001
LDH		229 (150–457)	184 (107–313)	<0.001
D Vit		12 (3.6–49)	16 (5.7–102)	<0.001
HbA1c (%)		9.39 (6.2–15)	5.5 (4.5–6)	<0.001
ESR (mm/h)		48 (1–127)	5 (2–51)	<0.001
WBC count (10^3^/uL)		9.8 (1.28–27)	7.7 (4.3–15)	0.015
NLR		4.06 (0.89–34.59)	1.99 (0.89–17.49)	<0.001
d-NLR		2.45 (0.15–10.34)	1.49 (0.45–3)	0.003
CRP (mg/L)		69 (0.8–387)	1.3 (0.1–36)	<0.001
CAR		2.33 (0.022–11.05)	0.03 (0.002–0.878)	<0.001
PCT (μg/L)		0.26 (0.01–4.7)	0.02 (0.01–0.07)	<0.001
Osteomyelitis n (%)		28 (48.3)	0	<0.001
PEDIS score 1 n (%)		5 (8.6)	0	<0.001
PEDIS score 2 n (%)		20 (34.5)	0	<0.001
PEDIS score 3 n (%)		25 (43.1)	0	<0.001
PEDIS score 4 n (%)		8 (13.8)	0	<0.001

Data are presented as mean ± SD or n (%), median, min-max. *p* < 0.05 is significant. CRP: C-reactive protein; CAR: CRP/albumin ratio; ESR: erythrocyte sedimentation rate; Hb: haemoglobin; LDH: lactate dehydrogenase; NLR: neutrophil-to-lymphocyte ratio; d-NLR: derived NLR; PCT: procalcitonin; PEDIS: Perfusion, Extent/Size, Depth/Tissue Loss, Infection, and Sensation; WBC: white blood cell.

**Table 2:** Comparison of clinical and laboratory parameters according to PEDIS score.

	PEDIS Score 1 (5)	PEDIS Score 2 (20)	PEDIS Score 3 (25)	PEDIS Score 4 (8)	*p*
Age (years)	71 (69–75)	62 (42–82)	60 (39–82)	64 (54–85)	0.08
BMI (kg/m^2^)	25 (22–28)	26 (22–36)	25 (21–35)	25 (22–40)	0.139
Duration of diabetes mellitus (years)	13.4 (8–20)	12.5 (0.5–30)	15.4 (4–33)	16.5 (12–20)	0.371
Duration of diabetic foot ulcer (days)	38.8 (7–90)	22.95 (4–60)	48 (4–360)	41.88 (15–120)	0.193
Osteomyelitis (n, %)	0	0	21 (80.8)	7 (87.5)	<0.001
Glucose (mg/dL)	136 (108–180)	176 (60–310)	208 (73–422)	188 (80–349)	0.372
HbA1c (%)	7.4 (6.4–8.5)	9.1 (6.2–13)	9.7 (6.3–15)	9.9 (7.8–12.6)	0.074
ESR (mm/h)	33 (7–59)	41 (2–102)	54 (1–127)	54 (6–77)	0.307
WBC count (10^3^/uL)	7.7 (3.8–11)	8.5 (1.28–14)	10.5 (2.5–27)	11.6 (4.5–24)	0.239
PCT (μg/L)	0.1 (0.05–0.02)	0.35 (0.01–4.7)	0.25 (0.06–1.8)	0.21 (0.1–0.5)	0.164
CRP (mg/L)	15.8 (0.8–51)	36.6 (1.1–159)	80.7 (1.1–387)	150 (17–360)	0.006
CAR	0.44 (0.02–1.41)	1.1 (0.024–4.18)	2.76 (0.026–11.05)	5.23 (0.47–9.47)	0.002
NLR	2.5 (1.22–4.24)	3.37 (0.89–6.33)	5 (1.35–34.59)	3.77 (1.3–8.94)	0.699
dNLR	1.58 (0.92–1.92)	2.29 (0.74–4.42)	2.81(0.87–10.34)	2.27 (0.15–5.49)	0.478

Data are presented as mean ± SD or n (%), median, min-max. *p* < 0.05 is significant. CRP: C-reactive protein; CAR; CRP/albumin ratio; ESR: erythrocyte sedimentation rate; NLR: neutrophil-to-lymphocyte ratio; dNLR: derived NLR; PCT: procalcitonin; PEDIS: Perfusion, Extent/Size, Depth/Tissue Loss, Infection, and Sensation; WBC: white blood cell.

**Table 3:** Correlations between the studied parameters.

	NLR		d-NLR		ESR		CRP		CAR		PCT		HBA1C		Osteomyelitis	
	r	*p*	r	*p*	r	*p*	r	*p*	r	*p*	r	*p*	r	*p*	r	*p*
d-NLR	0.819	<0.001														
ESR	0.310	0.001	0.419	<0.001												
CRP	0.448	<0.001	0.526	<0.001	0.582	<0.001										
CAR	0.445	<0.001	0.545	<0.001	0.618	<0.001	0.963	<0.001								
PCT	0.266	0.007	0.369	<0.001	0.122	0.221	0.126	0.206	0.120	0.228						
HbA1c	0.206	0.037	0.315	0.001	0.502	0.001	0.314	0.001	0.343	<0.001	0.226	0.022				
Osteomyelit	0.251	0.010	0.292	0.003	0.478	<0.001	0.516	<0.001	0.543	<0.001	0.117	0.238	0.538	<0.001		
PEDIS	0.240	0.015	0.348	<0.001	0.666	<0.001	0.579	<0.001	0.600	<0.001	0.212	0.032	0.781	<0.001	0.753	<0.001

r, Pearson’s correlation coefficient. *p* < 0.05 is significant.

## Data Availability

All data generated or analyzed during this study are included in this article. Further inquiries can be directed to the corresponding author.

## Abbreviations

AUC	Area Under the Curve
BMI	Body Mass Index
CAR	C-Reactive Protein to Albumin Ratio
CBC	Complete Blood Count
CRP	C-Reactive Protein
DFI	Diabetic Foot Infection
DM	Diabetes Mellitus
d-NLR	Derived Neutrophil-to-Lymphocyte Ratio
ESR	Erythrocyte Sedimentation Rate
HbA1c	Haemoglobin A1c
HPLC	High-Performance Liquid Chromatography
IQR	Interquartile Range
LDH	Lactate Dehydrogenase
NLR	Neutrophil-to-Lymphocyte Ratio
PCT	Procalcitonin
PEDIS	Perfusion, Extent/Size, Depth/Tissue Loss, Infection, Sensation
ROC	Receiver Operating Characteristic
SD	Standard Deviation
TNF-α	Tumour Necrosis Factor Alpha
WBC	White Blood Cell
